# Role of NLRP3 inflammasome in systemic sclerosis

**DOI:** 10.1186/s13075-022-02889-5

**Published:** 2022-08-16

**Authors:** Cong Lin, Zhixing Jiang, Ling Cao, Hejian Zou, Xiaoxia Zhu

**Affiliations:** 1 Division of Rheumatology, Huashan Hospital, Fudan University, 12 Wulumuqizhong Road, Shanghai, 200040 China; 2 Institute of Rheumatology, Immunology and Allergy, Fudan University, Shanghai, China

**Keywords:** Systemic sclerosis, NLRP3, Inflammasome, Caspase-1, IL-1β

## Abstract

Systemic sclerosis (SSc) is an autoimmune rheumatic disease with high mortality, which is featured by inflammation, vascular damage, and aggressive fibrosis. To date, the pathogenesis of SSc remains unclear and effective treatments are still under research. Active NLRP3 recruits downstream proteins such as ASC and caspase-1 and assembles into inflammasome, resulting in excretion of inflammatory cytokines including IL-1β and IL-18, as well as in pyroptosis mediated by gasdermin D. Various studies demonstrated that NLRP3 inflammasome might be involved in the mechanism of tenosynovitis, arthritis, fibrosis, and vascular damage. The pathophysiological changes might be due to the activation of proinflammatory Th2 cells, profibrotic M2 macrophages, B cells, fibroblasts, and endothelial cells. Here, we review the studies focused on NLRP3 inflammasome activation, its association with innate and adaptive immune cells, endothelium injury, and differentiation of fibroblasts in SSc. Furthermore, we summarize the prospect of therapy targeting NLRP3 pathway.

## Background

Systemic sclerosis (SSc) is an autoimmune disease characterized with inflammation, vascular damage, and fibrosis, in which the progressive fibrosis of skin and lungs is the most characteristic [1]. Epigenetics might play an important role in initiating the disease as many events were reported to trigger SSc onset [2]. However, the pathogenic mechanism of SSc still remains clarified. To date, there is lack of effective therapy for SSc. Pattern recognition receptors (PRRs) are cellular receptors which recognize various pathogen-associated molecular patterns (PAMPs) or damage-associated molecular patterns (DAMPs). NLRP3, a PRR which is classified as nucleotide-binding oligomerization domain (NOD)-like receptors (NLRs), is associated with numerous inflammation-related diseases [3]. Once activated, NLRP3 recruits downstream proteins and assembles into NLRP3 inflammasome, which leads to maturation and excretion of proinflammatory cytokines including interleukin(IL)-1β and IL-18, and exacerbates subsequent inflammatory cascades [4]. Recent studies suggest a remarkable link between NLRP3 and the pathological process in SSc, which might provide a potential therapeutic target for SSc treatment. In this paper, we summarize the key roles of NLRP3 in SSc pathogenesis, as well as the potential prospect of NLRP3 as a therapeutic target in SSc treatment.

## Overview of NLRP3

As the first line against invading microbes and cellular damage, innate immune cells could be activated by pathogens or defective cells through various PRRs expressed on membranes or in cytoplasm. PRRs expressed on membranes are transmembrane proteins, including Toll-like receptors (TLRs) and C-type lectin receptors (CLRs). The cytoplasmic PRRs consist of retinoic acid-inducible gene-I (RIG-I)-like receptors (RLRs), absent in melanoma 2 (AIM2)-like receptors (ALRs), and NLRs [5]. The heterotrimer NLRP3 is the most characterized protein in NLRs, formed by three domains: a C-terminal leucine-rich repeat (LRR) domain with self-regulating function, a nucleotide-binding NACHT domain with ATPase activity, because of which ATP binding is regarded necessary for NLRP3 activation [6], and a N-terminal pyrin domain (PYD) which recruits downstream proteins for assembling to functional form [6, 7]. Besides its remarkable ability in inflammatory regulation, NLRP3 exerts an indispensable role in embryonic development, antigen presentation, and cell death [8]. Once activated, NLRP3 proteins assemble into NLRP3 inflammasome consisted of a sensor (NLRP3), an adaptor (ASC, an apoptosis-associated speck-like protein containing a caspase recruitment domain), and an effector (caspase-1). Activated caspase-1 cleaves precursor of IL-1β and IL-18 into mature form to exert their biological effects. Abnormal activation of NLRP3 is considered as a key in several autoimmune diseases such as rheumatoid arthritis (RA), systemic lupus erythematosus (SLE), as well as SSc [3].

SSc, as an autoimmune disease featured by inflammation, vascular damage, and fibrosis, involves immune cells, endothelial cells (ECs), and fibroblasts [2]. Studies suggested that overactivation of NLRP3 might lead to inflammation and dysimmunity, acting as a sensor of danger signals [9, 10]. However, adverse evidence argues that NLRP3 appears to contribute to immune homeostasis [11, 12]. As a key role in vascular pathology, ECs could be activated by NLRP3, conducing to excretion of cytokines and further immune response [13]. NLRP3-mediated damage and necrosis of ECs were also found in diseases which are relevant to vasculopathy [14, 15]. NLRP3 also shows ability to activate fibroblasts, the most important cellular component of fibrosis, in chronic inflammatory diseases, inducing cell activation and differentiation [16, 17].

## Activation and regulation of NLRP3

The activation of NLRP3 requires two signals. The first one is the priming signal initiated by TLRs, regulating gene expression and posttranslational modification [7]. Once activated, TLRs recruits myeloid differentiation primary response gene-88 (MyD88) through homotypic interaction of its intracellular domain Toll-IL-1 receptor (TIR) domain and induces nuclear translocation of nuclear factor (NF)­κB, which upregulates the expression of NLRP3 and its downstream proinflammatory proteins including pro-caspase-1, pro-IL-1β, and pro-IL-18 [7]. The second signal is also called the activation signal which initiates the assembly of NLRP3 inflammasome after activated by stimuli, including virus infections, extracellular ATP, pore-forming bacterial toxins, crystals, particulate matters, and other inflammation-concerned signals [18, 19]. NLRP3 oligomerizes via NACHT domain interaction and then recruits its downstream proteins. Because of containing one PYD domain and one CARD domain, ASC could be recruited by NLRP3 through PYD-PYD homotypic interaction and aggregated into a macromolecular focus which is identified as ASC speck formation [20, 21]. ASC speck then gathers pro-caspase-1 via CARD-CARD homotypic interaction [20]. Polymerized pro-caspase-1 releases active caspase-1, which cleaves pro-IL-1β and pro-IL-18 into bioactive formation, further modulating inflammation [4, 22]. IL-1β is widely known as an efficient proinflammatory cytokine, which could mediate differentiation and infiltration of inflammatory cells and induce synthesis of more proinflammatory cytokines and the inflammatory change of microenvironment, contributing to enlargement of inflammation [23]. IL-18 was initially discovered to induce interferon (IFN)-gamma production. To date, previous studies have revealed pleiotropy of IL-18 in metabolism, allergic diseases, and autoimmune respond [24].

Pyroptosis is a recently discovered type of programmed cell death which could be conducted by activated NLRP3 inflammasome [25]. Different from apoptosis, pyroptosis leads to cell lysis and release of intracellular substances. Pore-forming protein gasdermin D (GSDMD) plays a crucial role in pyroptosis after being cleaved by caspase-1 [25]. GSDMD consists of N-terminal cell death domain (GSDMD^Nterm^), central linker region, and C-terminal self-inhibition domain [26, 27]. Once its C-terminal inhibitory region is cutoff by caspase-1, it embeds into cell membranes, forming a 10–14-nm channel which induces release of cytokines and pyroptosis [28].

Besides the canonical NLRP3 activation which promotes caspase-1 activation, the non-canonical NLRP3 activation contributes to activation of caspase-4 (or caspase-11 in mice) and caspase-5 [29]. Caspase-4 and -5 were reported to recognize LPS and then activate GSDMD [30, 31]. Evidence suggests that non-canonical activation might be an upstream event of canonical activation, as activated caspase-11 in non-canonical activation leads to caspase-1 activation and secretion of IL-1β and IL-18, which also requires NLRP3 inflammasome assembly [32, 33]. Although non-canonical activation of inflammasome is widely concerned as defense of Gram-negative bacteria, increased caspase-4 and caspase-11 in allergic airways present potential involvements in over-activated immune response [34, 35].

Different from typical PRRs, NLRP3 appears not to directly sense DAMP or PAMP. Although the mechanism remains to be fully elucidated, there are some studies trying to illuminate how NLRP3 inflammasome gets activated. Transmembrane pore proteins are supposed to play a crucial part in initiating inflammasome assembly. Stimuli like nigericin, mostly bacterial toxins, are pore-forming compounds, which might permeabilize cell membrane for ion flows while ion redistribution seems to be the key to inflammasome activation [36]. K^+^ efflux appears to be necessary for never in mitosis A-related kinase 7 (NEK7) activation and is confirmed to be an upstream event of NLRP3 activation [37, 38]. The mitotic kinase NEK7 is a newly found essential molecule for NLRP3 activation by directly combining to NLRP3 and mediating NLRP3 oligomerization [37, 39, 40]. A number of activation signals such as nigericin, crystals, and ATP induce K^+^ efflux before inflammasome activation, and the activation could be induced by low potassium independent of other triggers [41–43]. However, K^+^ concentration remains unchanged while NLRP3 activating in some research [44]. Other ion flows including Ca^2+^ influx and Cl^−^ efflux may also play a role in NLRP3 activation [45, 46]. But in contrary, evidence also shows that NLRP3 can be activated without Ca^2+^ influx or Cl^−^ efflux [41, 47]. It requires more evidence to clarify the role of ion flows in NLRP3 activation.

**Fig. 1:**
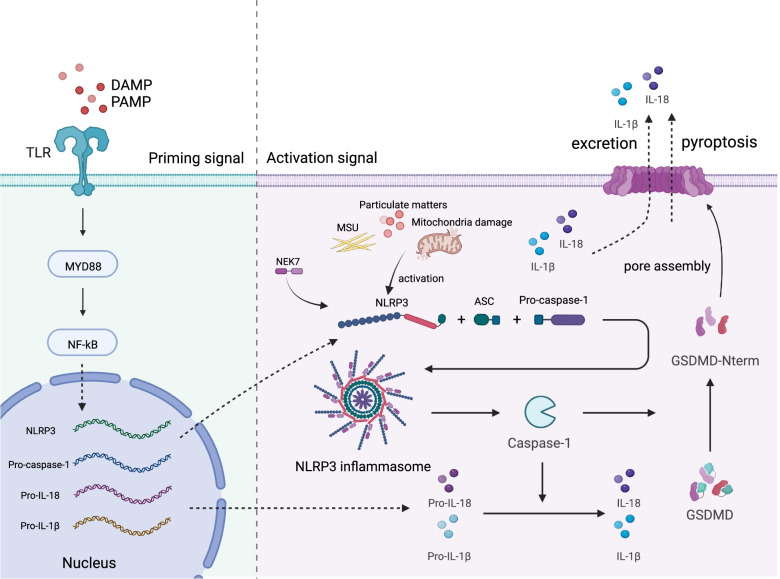
Activation of NLRP3 inflammasome. The activation of NLRP3 inflammasome requires two signals. Priming signal: TLRs are activated by extracellular DAMPs or PAMPs and mediate translocation of NF-κB, finally resulting in increased expression of NLRP3, pro-caspase-1, pro-IL-1β, and pro-IL-18. Activation signal: NLRP3 activated by stimuli like MSU and particulate matters activate, recruiting ASC and pro-caspase-1, then forming into inflammasome and releasing mature caspase-1. Caspase-1 cleaves pro-IL-1β, pro-IL-18, and GSDMD into mature form, contributing to further inflammation. TLR, Toll-like receptor; DAMP, Damage-associated molecular pattern; PAMP, Damage-associated molecular pattern; MSU, monosodium urate; GSDMD, Gasdermin D

Research indicates mitochondria damage may initiate NLRP3 activation by mitochondrial DNA (mtDNA) release and cardiolipin externalization [48]. Mitophagy may be a self-regulating mechanism to reduce NLRP3 inflammasome activation by mitochondrial dysfunction, and in which mitochondrial reactive oxygen species (mtROS) appears to be necessary [44, 49, 50]. Metabolism of glucose and fatty acid is suggested to show potential role on the inflammatory procedure. Hexokinase relocalization from mitochondrial membrane to cytoplasm is reported involving in NLRP3 activation [51]. Fatty acid might activate NLRP3 inflammasome through elevated mtROS via AMP-activated protein kinase signaling [52]. The contribution to inflammasome activation of extracellular ATP, hexokinase, and fatty acid indicate a possible association between energy reserve and NLRP3 activation. A summary of activation of NLRP3 could be found in Fig. 1.

## NLRP3 and SSc

Although what ignites the onset of disease remains an issue, immunology dysfunction and vascular damage seem to be early pathological changes in SSc, as perivascular edema and infiltration of immune cells are seen ahead of fibrotic involvement [53, 54]. Consistent inflammatory activation and autoimmune reaction might be responsible for differentiation of fibroblasts into myofibroblasts, the functional phenotype, which synthesize and excrete extracellular matrix (ECM) [55]. However, the exact pathogenesis of SSc is currently unknown. Except for genetics, environmental risk factors such as certain chemicals, viral infections, frequent thermal, and mechanical injury show great importance in the immune system dysregulation of SSc [56–58]. Abnormal activation and recruitment of immune cells, and consequent production of cytokines and autoantibodies are prominent in SSc pathological development. The consequent inflammation and over-deposition of ECM results in irreversible damage of tissue structure and ultimate organ failure, which is regarded as the tough nut in SSc treatments [55]. Previous research revealed that the expression of NLRP3 and its downstream proteins including caspase-1, IL-1β, and IL-18 increase in serum and skin biopsies from SSc patients and show positive relevance with lung and skin involvement [59, 60]. In bleomycin (BLM)-induced SSc mice, upregulation of IL-1β and IL-18 could be detected in serum and lung tissue, and alleviated disease performance could be found when NLRP3, ASC, or caspase-1 genes were knocked out [61, 62].

Circulating monocytes could differentiate into fibroblasts and macrophages [63], and both of them mediate disease progression of SSc [63–65]. As classical innate immune cells, macrophages are classified or polarized into two phenotypes, classical activated macrophages (M1) and alternative activated macrophages (M2). Traditional views regard M2 as the key role in fibrosis due to it producing profibrotic cytokines including IL-4, IL-13, and transforming growth factor β (TGF-β), leading to overexpression of ECM and scleroderma [63, 66]. Indeed, M1 also shows relevance to lung and skin involvement in SSc [67, 68]. NLRP3 inflammasome is usually regarded as an inhibitor in M2 activation; however, an increased number of M2 and the activation of NLRP3 inflammasome appear simultaneously in asthma patients [69]. What is more, research discovered that IL-1β-treated vascular endothelial cells mediated M2 activation in highly fibrotic skin [70].

Th1 and Th2 are two classical subtypes of T helper (Th) cell. Th1 secretes IFN-γ and tumor necrosis factor (TNF)-α, showing proinflammatory function [71], while Th2 secretes IL-4 and IL-13, presenting profibrotic properties [72]. It is comprehensible that NLRP3/IL-1 signaling activation regulating Th1/Th2 balance is involved in the immune mechanism in SSc [66, 73]. B cells participate in SSc pathogenesis through producing autoantibody, secretion of pro-fibrosis cytokines, and direct regulation of other effective cells like fibroblasts [74]. Dephosphorylase protein tyrosine phosphatase N22 (PTPN22) contributing to NLRP3 activation was reported as a crucial molecule in B cell signaling and autoantibody titer [75–77]. A recent study verified that BAFF, a B cell activating factor, activated NLRP3 in primary B cells and B lymphocyte lines through increasing NF-κB expression and upregulating ROS as well as K^+^ efflux [78]. NLRP3 displays capabilities in regulating antibody expression and cytokine secretion of B cells in infection model and plays potential role in inducing lymphocyte infiltration [79, 80]. These studies suggested that NLRP3/IL-1 signaling regulating T and B cells are related to the immune imbalance in SSc pathogenesis.

## NLRP3 and vascular damage in SSc

Microvascular dysfunction appears to play a prominent role at the early stage in SSc [81]. An increasing number of molecules inducing endothelial injury were proved NLRP3-dependent [82–85]. The high expression of NLRP3 in SSc skin biopsies is positively correlated with endothelin (ET)-1 expression [59]. Reduction of nitric oxide (NO), an efficient vasodilator, and increasing ET-1 expression appear to be responsible for vascular abnormalities like vasospasm, which is responsible for Raynaud’s phenomenon, a common symptom in SSc patients [54, 86]. NO also acts as an inhibitor of NLRP3 inflammasome assembly through removal of dysfunctional mitochondria while decreased NO might partly contribute to the activation of NLRP3 [87, 88].

ECs or endothelium injury gives rise to increased permeability as well as persistent vascular leakage and triggers the vascular microenvironment ischemia along with activating inflammation [58, 89]. ECs could be activated by IL-1β and exert its proinflammation action through secreting IL-1β in SSc vascular damage [81]. IL-1β participates in upregulating vascular endothelial growth factor (VEGF) and its receptor, resulting in hypoangiogenesis and vessel sparse in SSc patients [90]. Pulmonary hypertension (PAH) is related to severe outcomes in SSc patients. The expression of caspase-1 and IL-1β increases in peripheral blood mononuclear cells (PBMCs) of SSc patients with PAH compared to that in non-PAH SSc patients [91]. It suggests a potential function of IL-1β and its activator, NLRP3 inflammasome, in vasculopathy of SSc.

## NLRP3 and fibrosis in SSc

Excessive fibrosis is the most characteristic pathological feature in SSc, which results from excessive synthesis and deposition of ECM. The fibroblast-to-myofibroblast transition (FMT) after activation by inflammatory factors and mechanical damage is a crucial procedure in fibrosis. Myofibroblasts feature increased excretion of inflammatory factors and ECM [92]. ECM consists of collagen, elastin, glycosaminoglycans, tenascin, and fibronectin [58]. TGF-β is an important profibrotic mediator which could be secreted by several immune cells as well as myofibroblasts.

Recently, the role of NLRP3 inflammasome in SSc fibrosis has attached great concern. High expression of NLRP3, caspase-1, IL-1β, and IL-18 and over-activated NLRP3 inflammasome were found in skin biopsy specimen from SSc patients and showed a positive correlation with modified Rodnan skin thickness score (mRSS), a measure of skin involvement, in which higher scores represent severe skin fibrosis [59]. NLRP3 seems crucial in fibrosis as the involvement of skin and lung is alleviated when NLRP3 or ASC is knocked out in BLM-induced mice [62]. Similar results could be seen in caspase-1 or IL-18 knocked out mice [61]. Caspase-1 takes part in α-SMA expression of myofibroblasts from SSc dermis [62]. Reduction in collagen expression level and myofibroblast thickness in SSc derma could be induced by selectively blocking caspase-1 [62]. However, Bozena et al. found a decrease of serum caspase-1 in SSc patients comparing to healthy control and the caspase-1 levels demonstrate negative correlation with involvement in skin and visceral organ [93]. IL-18 was reported to upregulate IFN-γ and IL-13 production in T cells and induce lung fibrosis [94]. Zhang et al. also found increased αiSMA expression in myofibroblasts and enhanced fibrosis in liver, which could be inhibited by anti-IL-18 treatment [95]. It suggested that the function of NLRP3 inflammasome and its downstream products in SSc might be more than a proinflammatory role, which requires further investigation. Table 1 shows the role of NLRP3 inflammasome in fibrosis of SSc.

**Table 1:** Mechanism of NLRP3 inflammasome in SSc fibrosis

Samples	Method	Blockage target	Treatment	Results	Reference
SSc dermal and lung fibroblasts	Z-YVAD (OMe)-FMK	Caspase-1	-	Reduced expression of collagen, α-SMA, and contractile fibers	[62]
SSc dermal and lung fibroblasts	SiRNA of caspase-1	Caspase-1	-	Reduced secretion of hydroxyproline	[62]
C57BL/6 mice-derived fibroblasts	NLRP3−/−	NLRP3	BLM	Undetected hydroxyproline secretion	[62]
C57BL/6 mice-derived fibroblasts	ASC−/−	ASC	BLM	Undetected hydroxyproline secretion	[62]
C57BL/6 mice-derived fibroblasts	Z-YVAD (OMe)-FMK	Caspase-1	BLM	Reduced secretion of hydroxyproline	[62]
C57BL/6 mice	ASC−/−	ASC	BLM injection	Removed increased skin thickness and pulmonary fibrosis induced by BLM	[62]
C57BL/6 mice	NLRP3−/−	NLRP3	BLM injection	Removed increased skin thickness and pulmonary fibrosis induced by BLM	[62]
C57BL/6 mice	Caspase-1−/−	Caspase-1	BLM injection	Decreased neutrophils in BALF	[61]
C57BL/6 mice	IL-18−/−	IL-18	BLM injection	Decreased lung injury and pulmonary fibrosis	[61]
B6×129 hybrid mice	Caspase-1−/−	Caspase-1	BLM injection	Alleviated pulmonary fibrosis and lower lung hydroxyproline content	[61]

*Abbreviations*: *BLM* bleomycin, *BALF* bronchoalveolar lavage fluid, *SSc* systemic sclerosis

Recently, studies showed more interesting mechanisms of SSc onset. Fibroblasts from SSc patients exhibited higher Ca^2+^ permeability responding to P2X7R activating, which might give rise to NLRP3 activation in SSc [96]. MiR-155 was marked as the most expressed miRNA in fibroblasts from SSc patients and a necessary role in collagen synthesis [97, 98], and the function of miR-155 signaling in fibrosis is dependent on activation of NLRP3 [99].

## NLRP3 and musculoskeleton involvement in SSc

Musculoskeletal symptoms, involving joints, tendon, and muscle are quite common in SSc patients and contribute greatly in reducing quality of life [100, 101]. Articular involvement such as arthralgia and synovitis exists during course of SSc in nearly half of the patients and some of them are diagnosed as arthritis at first [102]. Myalgia and weakness resulted from myositis, while tendon friction rubs and tenosynovitis with a prevalence of about 11% are most featured tendon involvement in SSc [103]. Relations between NLRP3 and musculoskeletal involvement was reported as over-activated NLRP3 leads to chronic arthritis in mice [104]. Moreover, it was found that NLRP3 might contribute to ECM disorganization and inflammation in tendon injury [105]. What is more, upregulated expression of IL-1β and caspase-1 could be found in muscle biopsies from SSc patients with myositis [106]. Thus, the role of NLRP3 in musculoskeleton involvement deserves further investigation.

## Therapeutic target on NLRP3 in SSc

Recently, research of treatments aiming at NLRP3 inflammasome signaling cascade shows a promising target for inflammatory diseases including SSc. MCC950, also known as CP-456,773, is a highly specific NLRP3 inhibitor which declines maturation and excretion of IL-1β through suppressing assembly of NLRP3 inflammasome [107, 108]. Van et al. demonstrated NEK7 as a potential target of MCC950 whereas Perera et al. argued that MCC950 could not prevent NEK7-NLRP3 interaction [109, 110]. MCC950 has shown its protective property through inhibition of NLRP3 activation in silicon dioxide-induced pulmonary fibrosis through preventing macrophage-derived IL-1β excretion and then impeding myofibroblasts transition [111]. The anti-fibrosis function of MCC950 is also reported in liver, myocardium, and kidney [112–114]. However, MCC950 presented hepatotoxicity in clinical trials for RA, which might limit its further development [115]. CY-09 is a newly found selective NLRP3 inhibitor which inhibits ATPase activity through directly binding to NACHT domain [116]. Gao et al. suggested that CY-09 could reduce myocardial fibrosis induced by ischemia [117].

Addition to small-molecule inhibitors, several endogenous molecules targeting NLRP3 are also of note. A20, an anti-inflammatory protein which is also known as TNF-α-induced protein 3 (TNFAIP3), shows a protective role in pulmonary fibrosis and it was reported to reduce NLRP3-mediated cytokine secretion and pyroptosis in RA [104, 118]. What is more, β-hydroxybutyrate (BHB), an oxidative metabolite of fat, was demonstrated to suppress NLRP3 inflammasome activation through inhibiting K^+^ efflux and ASC speck formation [119]. Intriguingly, ketogenic diet, which could elevate BHB levels in serum, also reduces caspase-1 maturation and IL-1β secretion, indicating a supplementary means of dietary therapy to treatment in NLRP3-relevant diseases [119].

Caspase-1 as the executor in NLRP3 inflammasome is also a remarkable target for NLRP3 block. YVAD-CHO is a reversible caspase-1 inhibitor, which reduces IL-1β excretion in SSc monocytes [120]. Treatment of another caspase-1 inhibitor Z-YVAD-FMK eliminates oversecretion of IL-1β in fibroblasts from skin and lung of SSc patients [62]. IL-1 signaling blockers targeting IL-1R have attracted much attention as several of them have been used in clinic. Anakinra, an IL-1R antagonist, manifests capacity in improving BLM-induced pulmonary fibrosis in mice [121]. Evidence in clinical trial conducted in RA patients suggests that anakinra improves vascular function [122]. However, no clear conclusion could be drawn in SSc management due to the lack of data. A summary of NLRP3 inflammasome inhibitors is shown in Table 2.

**Table 2:** Inhibitors of NLRP3 inflammasome in SSc therapy

Inhibitor	Target	Evidence	Reference
MCC950(CP-456,773)	NLRP3	Prevents IL-1β excretion, impeding myofibroblast transition and pulmonary fibrosis	[111]
		Reduces inflammation and liver fibrosis	[112]
		Reduces immune cell infiltration and myocardial fibrosis	[113]
		Reduces immune cell infiltration and collagen production in kidney	[114]
CY-09	NLRP3	Reduces ischemia-induced myocardial fibrosis	[117]
A20 (TNFAIP3)	NLRP3	Prevents pulmonary fibrosis	[118]
		Reduces NLRP3-mediated cytokine secretion and pyroptosis in RA	[104]
BHB	NLRP3	Reduces caspase-1 maturation and IL-1β secretion	[119]
YVAD-CHO	Caspase-1	Reduces IL-1β excretion in SSc monocytes	[120]
Z-YVAD-FMK	Caspase-1	Eliminates oversecretion of IL-1β in fibroblasts from skin and lung of SSc patients	[62]
Anakinra	IL-1R	Alleviates BLM-induced inflammation and pulmonary fibrosis	[121]
		Improves vascular function in RA patients	[122]

*Abbreviations*: *SSc* systemic sclerosis, *IL* interleukin, *RA* rheumatoid arthritis, *BHB* β-hydroxybutyrate

Possible side effects should be of note as NLRP3 plays an important role in immune defense system. Treatment with MCC950 weakens early protective immune response towards influenza A virus [123]. Infections like pneumonia and gastroenteritis turns out to be the most common serious adverse events in anakinra treatments [124]. Thus, it requires cautious consideration when taking NLRP3 and its products as the therapeutic target.

## Conclusion

As its function on mediating proinflammatory cytokines such as IL-1β and IL-18, NLRP3 is involved in many inflammatory immune-relevant diseases, including SSc. This review elaborate NLRP3 and its potential association with the autoimmune dysfunction, vascular damage, fibrosis of skin, and visceral organ in SSc. Despite contradictory conclusions, NLRP3 might play a potential role in SSc pathogenesis and offers promising insight into SSc treatment.

## Data Availability

All data are available from the corresponding author.

## Abbreviations

SSc: Systemic sclerosis
PRR: Pattern recognition receptors
PAMP: Pathogen-associated molecular pattern
DAMP: Damage-associated molecular pattern
NLR: Nucleotide-binding oligomerization domain-like receptor
IL: Interleukin
TLR: Toll-like receptor
CLR: C-type lectin receptor
RLR: Retinoic acid-inducible gene-I-like receptor
ALR: Absent in melanoma 2-like receptor
LRR: Leucine-rich repeat
PYD: Pyrin domain
IFN: Interferon
RA: Rheumatoid arthritis
SLE: Systemic lupus erythematosus
MyD88: Myeloid differentiation primary response gene-88
TIR: Toll-IL-1 receptor
GSDMD: Gasdermin D
Nterm: N-terminal cell death domain
mtDNA: Mitochondrial DNA
mtROS: Mitochondrial reactive oxygen species
NEK7: Never in mitosis A-related kinase 7
ECM: Excrete extracellular matrix
BLM: Bleomycin
M1: Classical activated macrophages
M2: Alternative activated macrophage
TGF-β: Transforming growth factor β
Th: T helper
TNF: Tumor necrosis factor
PTPN22: Protein tyrosine phosphatase N22
EC: Endothelial cell
VEGF: Vascular endothelial growth factor
PAH: Pulmonary hypertension
PBMC: Peripheral blood mononuclear cell
ET: Endothelin
NO: Nitric oxide
FMT: Fibroblast-to-myofibroblast transition
mRSS: Modified Rodnan skin thickness score
TNFAIP3: TNF-α-induced protein 3
BHB: β-hydroxybutyrate
